# Endoplasmic Reticulum Stress and Oxidative Stress: A Vicious Nexus Implicated in Bowel Disease Pathophysiology

**DOI:** 10.3390/ijms18040771

**Published:** 2017-04-05

**Authors:** Wai Chin Chong, Madhur D. Shastri, Rajaraman Eri

**Affiliations:** School of Health Science, University of Tasmania, Newnham TAS 7248, Australia; chongwc1993@gmail.com

**Keywords:** endoplasmic reticulum stress, unfolded protein response, oxidative stress, antioxidant mechanisms, misfolded protein, inflammatory bowel disease

## Abstract

The endoplasmic reticulum (ER) is a complex protein folding and trafficking organelle. Alteration and discrepancy in the endoplasmic reticulum environment can affect the protein folding process and hence, can result in the production of misfolded proteins. The accumulation of misfolded proteins causes cellular damage and elicits endoplasmic reticulum stress. Under such stress conditions, cells exhibit reduced functional synthesis, and will undergo apoptosis if the stress is prolonged. To resolve the ER stress, cells trigger an intrinsic mechanism called an unfolded protein response (UPR). UPR is an adaptive signaling process that triggers multiple pathways through the endoplasmic reticulum transmembrane transducers, to reduce and remove misfolded proteins and improve the protein folding mechanism, in order to improve and maintain endoplasmic reticulum homeostasis. An increasing number of studies support the view that oxidative stress has a strong connection with ER stress. During the protein folding process, reactive oxygen species are produced as by-products, leading to impaired reduction-oxidation (redox) balance conferring oxidative stress. As the protein folding process is dependent on redox homeostasis, the oxidative stress can disrupt the protein folding mechanism and enhance the production of misfolded proteins, causing further ER stress. It is proposed that endoplasmic reticulum stress and oxidative stress together play significant roles in the pathophysiology of bowel diseases.

## 1. Introduction

Endoplasmic reticulum (ER) is a vital cellular organelle in eukaryotes. It serves an important role in protein biosynthesis and post-translational modification processes. After transcription, the messenger ribonucleic acids are translocated to ER for translation, to produce a nascent protein [1]. Post-translational processes, such as disulfide bonding and *N*-glycosylation, produce mature, unique, and functional proteins [1]. Any discrepancy in the endoplasmic reticulum has an adverse impact on the protein biosynthesis and modification process, resulting in the production of misfolded proteins. Most misfolded proteins do not have a particular function in the cellular metabolism. Therefore, they are subjected to either refolding or degradation [1].

ER stress (ERS) arises after the accumulation of misfolded proteins in the ER. ERS can reduce the production of functional proteins and even lead to apoptosis. To relieve the cell from ERS, an evolutionarily conserved mechanism called the unfolded protein response (UPR) is activated [2]. UPR consists of numerous complex, multifactorial, and parallel pathways that act to reduce and remove misfolded proteins. UPR triggers multiple mechanisms to decrease protein synthesis, enhance the protein folding mechanism, and remove the terminal misfolded protein [2,3].

ERS has been linked to oxidative stress (OS) in the pathophysiology of numerous diseases. OS is a form of cellular damage caused by excess production and an accumulation of reactive oxygen species (ROS) that overwhelm the already compromised antioxidant defence mechanism. ROS are free radicals that are produced as a by-product of cellular metabolism. A number of biological disorders and external agents are known to upregulate the production of ROS. Recent studies show that OS can elicit a reduction-oxidation (redox) imbalance and worsen ERS through reducing the efficiency of protein folding pathways and increasing the production of misfolded proteins [4].

In the present review, we summarize our current knowledge on the correlation between the ERS and OS, as well as the signaling mechanisms triggered in response to both ERS and OS.

## 2. Endoplasmic Reticulum

ER is the major organelle in eukaryotes that is responsible for protein biosynthesis and modification, including the translation of messenger ribonucleic acid (mRNA), protein glycosylation, disulfide bonding, and post-translational modifications [5]. Besides protein biosynthesis, ER also plays a significant role in calcium storage, lipid biosynthesis, detoxification, and energy metabolism, as well as nucleus-cytosol signaling [6,7].

**Figure 1:**
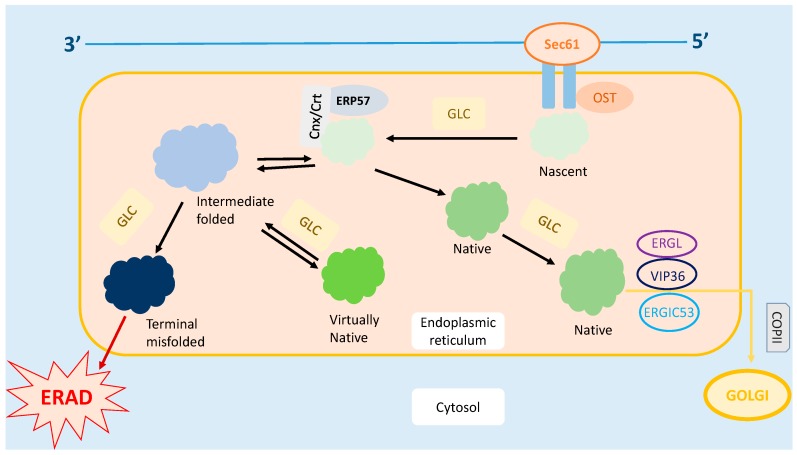
Protein trafficking in ER. Post mRNA translation, the nascent protein is transferred into the ER via the Sec61 channel, followed by the OST-mediated addition of *N*-acetylglucosamine residue on the protein. Glucose present on the oligosaccharide core undergoes glycosylation. The protein is then folded, and the disulfide bond is formed within the protein to stabilize its structure. Further, the correctly folded protein is cleaved and transported to the Golgi apparatus for further modifications. On the other hand, the misfolded protein rebinds to Cnx/Crt and is subjected to the refolding process. However, if the protein is recognized as terminally misfolded, it will be removed from the ER and subjected to ER-Associated Degradation (ERAD). * Cnx, Calnexin; COPII, Coat Protein II; Crt, Calreticulin; EDEM, ER Degradation Enhancer- α-Mannosidase; ERGIC53, ER-Golgi Intermediate Compartment 53 kDa Protein; ERGL, ERGIC53-like Protein; Erp57, ER-located PDI 57 kDa Protein; GlcI, Glucosidase I; GlcII, Glucosidase II; OST, Oligosaccharide transferase; UGTI, Uridine Diphosphate Glucose:glycoprotein Glycosyltransferase; VIP36, Vesicular Integral-membrane Protein 36.

ER is also responsible for the quality control of the proteins produced. Several complex mechanisms tightly regulate the protein trafficking in the ER [8], as shown in Figure 1. Under these quality control mechanisms, only correctly folded proteins can be transported out of the ER, while the misfolded proteins are either refolded or undergo ER-Associated Degradation (ERAD) [8,9,10]. Therefore, it is crucial for the ER to differentiate the misfolded proteins from those which are correctly folded and nascent. Nascent proteins display an addition of *N*-acetylglucosamine-mannose-glucose [11]. Only correctly folded proteins undergo glucosidase cleavage and are translocated to the Golgi complex [8,10]. Meanwhile, misfolded proteins are recognized by their abnormal hydrophobic bonds via the chaperones that reside inside the ER [8]. These misfolded proteins are prevented from exiting the ER by Calnexin/Calreticulin (Cnx/Crt) [8].

To ascertain that functional and mature proteins are being produced, the ER contains several molecular chaperones that support protein biosynthesis. These chaperones include Heat Shock Proteins (HSP) such as HSP33, HSP60, HSP70, HSP90, and others. HSP33 holds and prevents protein aggregation and Protein Disulfide Isomerase (PDI), which regulates disulfide bonds within a polypeptide [2,8,12,13]. These chaperones play a fundamental role in maintaining ER homeostasis. Studies have shown that an interruption in the functional properties of chaperones can cause significant impacts on cell homeostasis. For instance, the in vitro chemical inhibition of chaperones (using tunicamycin) results in the inhibition of *N*-glycosylation activity in the protein, causing an accumulation of misfolded proteins [8]. It must be noted that the protein and gene expression of these chaperones demonstrate a significant elevation when the cells contain misfolded proteins in the ER [8]. Therefore, such chaperones are considered as markers for the misfolded protein assays [8].

## 3. Endoplasmic Reticulum Stress

The protein folding process in the ER is crucial and acutely sensitive to intracellular and extracellular stimuli, such as the ER calcium ion, energy storage and redox homeostasis, elevation in mRNA translation, cytotoxicity, and inflammation [14,15,16]. A few misfolded proteins are commonly found in the ER [2,17]. Numerous studies have demonstrated that any interruption in the protein biosynthesis, such as an abnormal elevation in the protein biosynthesis, inhibition of the disulfide bond formation, metabolic energy depletion, and perturbation in *N*-glycosylation, can lead to the formation of a misfolded protein [2,17]. Importantly, the protein misfolding rate is directly proportional to the complexity and amount of the protein synthesized [8,17]. For instance, goblet cells have a higher amount of misfolded proteins and are more susceptible to the accumulation of misfolded proteins compared to other cells, since goblet cells secrete an abundant amount of complex mucin MUC2 protein [8,17]. In normal conditions, the ER has an advanced protein quality control mechanism to refold and remove the misfolded protein [8,17,18]. However, ER homeostasis can still be compromised if the protein misfolding rate exceeds the normal threshold of refolding, causing the accumulation of misfolded proteins, and leading to an unusual phenomenon known as ERS.

**Figure 2:**
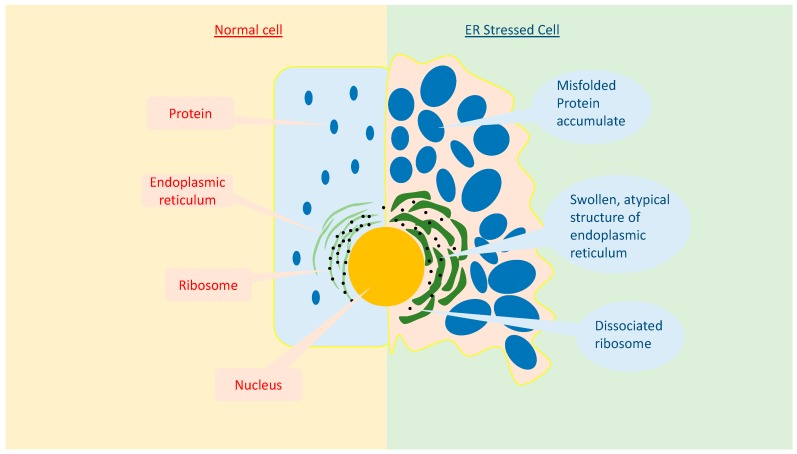
Comparison between a normal and ER stressed cell. Compared to the normal cell, the ER stressed cell exhibits a significant difference in the ER structure, including luminal swelling and a dissociated ribosome.

ERS not only affects cellular homeostasis, but also cellular morphology. As shown in Figure 2, cells that undergo ERS have an altered ER morphology, such as luminal swelling and atypical structures [19]. ERS has an enormous impact on overall cellular processes. It is known to reduce the functional transcription and translation processes, as well as the intracellular and extracellular signaling pathways [6]. As a result, it can lead to various diseases [6].

## 4. Unfolded Protein Response (UPR)

To help resolve ERS, cells develop a network of parallel, independent, and multifactorial signaling and transcriptional pathways known as the UPR. The UPR consists of numerous transcription factors and enzymes, which have been successfully discovered and well-studied over several decades [6,20,21]. UPR is aimed at relieving the ERS by decreasing the translation and transcription of general proteins, accelerating the ER protein folding and refolding ability, and enhancing ERAD to remove and reduce the accumulation of misfolded proteins in the ER. However, if the stress condition is too severe, or the UPR is impaired, then the UPR fails to alleviate the misfolded protein. As a consequence, the apoptotic signaling mechanism is triggered [6,20].

**Figure 3:**
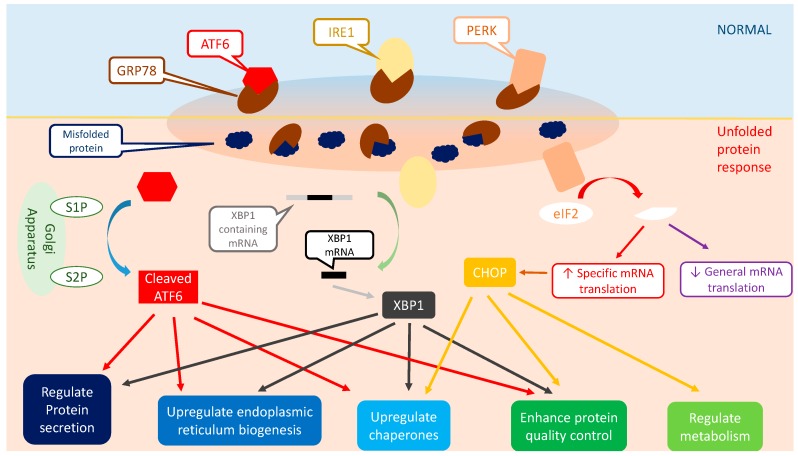
The UPR downstream signaling pathway. The UPR signal transducers, PERK, IRE1, and ATF6, are bounded by GRP78 under the normal condition. The accumulation of misfolded proteins triggers GRP78 to release and activate the UPR signal transducers to bind to the misfolded proteins, hence activating the UPR downstream signaling pathways. In the IRE1 pathway, IRE1 cleaves and releases XBP1 mRNA, which is translated to the active XBP1 protein that enhances the expression of UPR target genes. Meanwhile, PERK phosphorylates eIF2 and inhibits general mRNA translation, while upregulating the gene expression of resident ER proteins, hence reducing the ER workload to relieve the ERS. On the other hand, ATF6 migrates to the Golgi apparatus and is cleaved by the S1P and S2P protein, to expose its bZIP domain. Activated ATF6 is translocated to the nucleus and acts as a transcription factor to enhance the gene expressions of several ER resident proteins that contribute to protein folding, secretion, modification, and ERAD processes. * bZIP, Basic Leucine Zipper; eIF2, Eukaryotic Initiation Factor 2; ERAD, ER-associated Degradation; S1P, Sphingosine-1-Phosphate; S2P, Sphingosine-2-Phosphate.

The UPR process has a critical major player known as Glucose Regulated Protein 78 (GRP78). GRP78 plays a pivotal role in the quality control of protein biosynthesis, including protein synthesis, folding, and assembly [18]. Additionally, GRP78 acts as an initiator of the UPR signaling pathway. It is established that there are three ER-localized transmembrane signal transducers that initiate the UPR [22,23,24]. These transducers are known as Inositol Requiring Kinase 1 (IRE1), Protein Kinase-like ER Kinase (PERK), and Activating Transcription Factor 6 (ATF6) [22,23,24]. These are constitutively expressed in the cells, but are tightly bounded by GRP78, and hence, are inactive under normal conditions [8,25,26]. Once misfolded proteins are formed in the ER, GRP78 unbinds and releases these transducers to locate and bind the misfolded proteins [26]. This event activates the transducers and results in the UPR downstream signaling pathways [26,27], as depicted in Figure 3.

### 4.1. Inositol-Requiring Protein 1 (IRE1)

IRE1 is an endoribonuclease that is localized in the ER membrane. It is the first component of the UPR signaling transducer that was discovered in Saccharomyces cerevisiae in 1990 via genetic screening in the UPR signaling model, and was found to be a proximal sensor of the UPR [28,29,30]. It is also the most conserved ERS transducer [28,29]. Recently, two isoforms of IRE1 have been identified, named IRE1α and IRE1β, and are encoded by differences in their genome and expression profiles [8,18]. Among these isoforms, IRE1α is highly conserved and constitutively expressed in eukaryotic cells [18].

IRE1 contains a Ser/Thr kinase domain and an endoribonuclease domain in its cytosolic part. As depicted in Figure 3, in a stress-free environment, IRE1 remains in an inactivated form due to the association between its cytoplasmic endoribonuclease domain and GRP78 [8,31]. In ERS, the dissociation of GRP78 from IRE1, and the subsequent binding of GRP78 to the misfolded proteins, results in an indirect activation of IRE1 [32,33]. Moreover, specific homodimerization and auto-phosphorylation processes play a pivotal role in the activation of IRE1. The endonuclease activity of activated IRE1 cleaves a 26 bp intron from mRNA, encoding a specific basic leucine zipper-containing transcription factor, also known as X-Box Binding Protein-1 (XBP1) [6,34,35]. These events trigger a translational frameshift, leading to the production of activated forms of the XBP1 protein [6,34,35]. Activated XBP1 is a crucial transcriptional factor required for the activation of numerous UPR target genes that are known to regulate ER protein folding, ERAD, ER membrane expansion, and phospholipid synthesis, as well as protein intracellular-extracellular translocation [14]. XBP1 directly or indirectly binds to several transcriptional factors associated with the UPR, resulting in an increased expression of the target genes [8,18]. These target genes are involved in the ERAD process; for instance, an in vitro study determined that stressed cells with mutated IRE1 or XBP1 release displayed defective ERAD [36].

The role of IRE1 in the activation of the inflammation and apoptotic signaling pathways is well-established. Under ERS, IRE1 cleaves several ER-localized mRNAs, along with their RNase domain, via a process called the Regulated IRE1-dependent Decay of mRNA [14,37]. It is hypothesised that the IRE1-induced loss of ER-localized mRNAs triggers inflammation and apoptotic downstream signaling, either via Tumour Necrosis Factor Alpha Receptor-associated Factor-2 (TRAF2) or the c-Jun N-Terminal Kinase (JNK) pathway [14,37]. Moreover, IRE1 is also imperative in regulating the homeostasis of intestinal epithelial cells. However, the precise mechanisms underlying the regulation of homeostasis by IRE1 still remains obscure. A recent study by Zhang et al. found that IRE1 knockout mice have a markedly reduced number of goblet cells and were highly susceptible to dextran sulfate sodium-induced colitis [38]. Overall, the above findings demonstrate the potential importance of IRE1/XBP1 pathways during the UPR and ER quality control mechanisms.

### 4.2. Protein Kinase-Like Endoplasmic Reticulum Kinase (PERK)

PERK, a type-1 ER-associated transmembrane protein, manages serine/threonine protein kinase activity via its cytoplasmic domain [6]. Similar to IRE1, it is attached to GRP78, and therefore remains in an inactivated mode under the stress-free condition (Figure 3) [39]. During the presence of misfolded proteins, PERK is released and activated via dimerization and trans- auto-phosphorylation [25,40]. Activated PERK has a catalytic domain that shares a substantial homology with a heterotrimeric protein known as Eukaryotic Translational Initiation Factor 2 on α Subunit at Ser51 (eIF2α) [25,40]. The eIF2α is involved in the protein translation process via its reaction with methionyl-transfer Ribonucleic Acid (Met-tRNA) and Guanosine Triphosphate, resulting in the formation of the eIF2α-GTP-Met-tRNA complex [25,40,41]. This complex is required for the AUG initiation codon to recognize and bind the 60S ribosomal subunit during mRNA translation [25,42]. The phosphorylation of eIF2α enables PERK to attenuate mRNA translation, resulting in an abrogation of the protein synthesis and reduction in the ER workload [43,44]. Although eIF2α phosphorylation attenuates the protein synthesis, phosphorylated eIF2α is vital for the mRNA translation for Activating Transcription Factor 4 (ATF4) [25,42]. ATF4 is critical for the UPR downstream signaling. The 5′-untranslated region of ATF4 mRNA possesses regulators of mRNA translation, known as the Upstream Open Reading Frame (uORF), and the translation of uORFs leads to the missense mutation and attenuation of ATF4 translation [45,46]. However, during the UPR, eIF2α phosphorylation inhibits uORF, hence permitting the translation of ATF4 [45,46]. Based on scientific evidence, ATF4, PERK, and eIF2α phosphorylation are essential for the expression of genes that encode protein translocation and synthesis, an anti-oxidative response, and apoptosis. [45,46]. Examples include Growth Arrest and DNA Damage 34 and the CAAT enhancer binding homologous protein.

Interestingly, recent studies have revealed that other cytosolic kinases also phosphorylate eIF2α at Ser51 under stress conditions. For instance, General Control Non-Repressed 2 Kinase is activated by amino acid deficiency, Protein Kinase is stimulated by double-stranded RNA (dsRNA), and heme-regulated eIF2α Kinase is initiated by heme-deprivation [14,47]. The initiation and regulation of such mRNA translation processes via eIF2α kinase are collectively known as integrated stress responses [14,47].

### 4.3. Activating Transcription Factor 6 (ATF6)

ATF6, the 90 kDa protein containing a transmembrane domain, is one of the UPR transducers [48]. Under the stress-free condition, it is localized to the ER membrane and bounded by GRP78 [8,48]. There are two distinct ATF6 isoforms in mammalian cells, ATF6α and ATF6β [49]. ATF6α plays a vital role in optimizing the protein folding process and ERAD [49,50]. It was shown that ATF6α knockout mice were more vulnerable to ERS compared to wild-type mice, while mutated ATF6β mice displayed no significant effect towards ERS [49,50]. On the other hand, the double knockout of ATF6α and ATF6β caused embryonic lethality in mice; although the actual reason for this remains to be identified [14].

Unlike IRE1 and PERK, ATF6, following its dissociation with GRP78, gets transported to the Golgi apparatus in response to the ERS (Figure 3) [6]. The N-terminal domain of ATF6 is cleaved by sphingosine-1-phosphate and sphingosine-2-phosphate through the regulated intramembrane proteolysis, forming an active ATF6 transcription factor in the cytoplasm [6,51] [24]. The active ATF6 translocates to the nucleus and binds to the CAAT Binding Protein and ERS response element 1, contributing to the upregulation of the target gene expression and the ER client protein synthesis [24,52]. These ER client proteins include GRP78, ER Protein 99, and DnaJ HSP family member C3 [14]. The involvement of ATF6α is crucial in relieving ERS due to its ability to improve protein folding, post-translational modification, and trafficking [14]. Cao et al. reported that during chronic ERS, ATF6α deficient mice have significantly higher mortality rates compared to the wild-type mice [14]. Interestingly, ER-stressed transgenic ATF6α knockout mice exhibit an increased expression of common ERS genes, like GRP78, but did not upregulate the production of XBP1 and ATF4, suggesting the potential role of ATF6α in the regulation of XBP1 and ATF4 [53].

## 5. Protein Folding Challenge in Intestinal Secretory Cells

Secretory cells have a higher risk of ERS compared to other cell types, as they constantly produce plenty of proteins. The secretory cells include plasma-and pancreatic β-cells, which manufacture many antibodies and the insulin hormone. The heavy burden of protein synthesis is compensated for by these secretory cells via advanced protein regulatory mechanisms, which optimize protein folding and the elimination of misfolded proteins [1,54]. In the intestinal tract, secretory cells produce complex proteins to maintain optimal intestinal conditions. One example of this is goblet cells, which produce mucin glycoproteins, forming the mucosal barrier around the intestinal tract lumen, thus defending the intestinal epithelial lining from luminal microbial flora [8]. Goblet cells are mainly located throughout the intestine (small and large), from the duodenum to the rectum. Their synthesis products, mucin glycoproteins, are complex proteins containing disulfide-linked homo-oligomers with more than 5000 amino acids [55]. The production of mucin glycoproteins is elevated by toxins and nervous stimuli [56,57]. There are several types of mucin glycoproteins produced by the goblet cells, including mucin-type II (MUC2), MUC5AC, and MUC6 [55,58,59]. MUC2 is the most abundant type of mucin glycoprotein and is found throughout the intestinal tract [55,58,59,60]. It has a central repetitive region enriched with proline, serine, and threonine residues, that undergoes extensive glycosylation and post-translational modifications during the protein folding process [58,59]. Despite the complexity of MUC2 synthesis, it is constitutively secreted in high amounts, forming the major macromolecular component of the mucosal barrier. Besides MUC2, goblet cells also produce other proteins, such as Trefoil Factor 1 (TFF1), the Fc Fragment of Immunoglobulin G Binding Protein (FCGBP), and Resistin-like Molecule β (RELMβ) [61]. The high demand of protein synthesis imposes a considerable burden on the goblet cells, hence making them more susceptible to the ERS.

Recent studies utilizing mouse models document the potential role played by the goblet cells during intestinal inflammation and ERS. Although multiple studies have been conducted so far, the correlation between ERS, goblet cells, and inflammation remains obscure. MUC2 deficient mice were reported to have spontaneous colitis and were more susceptible to dextran sulfate sodium-induced colitis [62,63]. On the other hand, Gfi-deficient, as well as diphtheria toxin transgenic mice models with no functional goblet cells, were not associated with spontaneous inflammation, suggesting a correlation between the goblet cells and the release of pro-inflammatory cytokines, leading to inflammation [64,65]. As a shred of evidence, Winnie and Eeyore mouse models that have abnormalities in functional MUC2 production (due to point mutations in the *MUC2* gene), exhibit spontaneous inflammation [56,66]. Although the ERS response is enhanced in these mouse models, it is yet to be confirmed whether the ERS response is caused by the spontaneous inflammation, is due to a malfunction in MUC2 glycoprotein synthesis, or is derived from both of these events [67]. Conversely, in the clinical studies, ERS was observed to play a significant role in the intestinal inflammation. Intestinal biopsy samples obtained from patients suffering from ulcerative colitis and Crohn’s disease had an elevated expression of XBP1 [67,68]. Furthermore, polymorphism in the *XBP1* gene was also detected in both of the disorders. Three single nucleotide polymorphisms have been discovered in patients with ulcerative colitis and Crohn’s disease, compared to normal individuals [67,68]. In Vitro studies revealed that these single nucleotide polymorphisms reduce the activation of XBP1 and other UPR target genes [67,68]. Taken together, goblet cells play a critical part during ERS and intestinal inflammation; however, further studies are warranted to confirm this hypothesis.

Similar to the goblet cells, paneth cells are also highly susceptible to the ERS. Paneth cells are intestinal epithelial cells that form the lining of the small intestine and are also located at the base of intestinal crypts. In the histological examination, these cells can be identified by their large eosinophilic refractile granule that occupies most of the space in the cytoplasm. Paneth cells are important in defending the intestinal tract via the production of antimicrobial proteins to eliminate gastrointestinal pathogens and maintain homeostasis between the host and the microbe. Interestingly, Bertolotti et al. reported that UPR plays a vital role in the maintenance of homeostasis in paneth cells [69]. It was observed that mice with depleted Endoplasmic Reticulum to the Nuclear Signalling 2 (ERN2) gene had little to no IRE1β protein expression [69]. Unlike IRE1α, which are expressed ubiquitously, IRE1β are only expressed in the intestinal or lung epithelial cells [69]. These mutated mice were susceptible to dextran sodium sulfate- induced colitis compared to the wild-type [69]. The study demonstrated the influence of UPR pathways in maintaining homeostasis on the intestinal interface when exposed to environmental microbes [69]. Furthermore, the significance of UPR in the development of paneth cells was also found in a transgenic mouse model with XBP1 deletion [68]. In mice with an XBP1 deficiency, the development of paneth cells was completely seized and distorted [68]. Microscopic evaluation revealed a dramatic disorganization of the intestinal architecture, with a complete loss of functional paneth cells in the intestinal tract [68]. Moreover, there was a decline in the number of goblet cells in the intestinal tract [68]. These mice were also found to be highly susceptible to colitogenic stimuli and microbiota-induced enteritis [68]. Overall, XBP1 perturbations can seize the development of paneth cells, and thus compromise the immunological protection of the intestinal tract.

## 6. Endoplasmic Reticulum Stress and Autophagy

Autophagy is a naturally regulated, destructive cellular process that disassembles unwanted or dysfunctional components of the body. It coordinates the degradation of organelles, proteins, and lipids within the cells, to maintain the homeostatic function. Physiological perturbations include starvation, ERS, and infection. During autophagy, intracellular debris and organelles are surrounded and degraded by lysosomes [70]. Studies have proposed a substantial link between autophagy, UPR, and ERS [71,72,73,74]. As mentioned earlier, the accumulation of unfolded and misfolded proteins during ERS enforces a massive workload on the cells, especially secretory cells, which are known to produce enormous quantities of proteins [75]. Interestingly, autophagy activities are also high in paneth cells, and are thought to be useful for uncontrolled ERS [76,77]. Adolph et al. reported that Autophagy Related Protein 16-1 (ATG16L1) deficient molecules exhibit discontinuous transmural ileitis similar to the symptom observed in Crohn’s disease after an ERS challenge [76]. Additionally, under prolonged ERS, UPR triggers the release of Ca^2+^, initiating the activation of Calcium/Calmodulin-dependent Protein Kinase Kinase β (CaMKKβ) and 5′ Adenosine Monophosphate activated Protein Kinase (AMPK) [78,79]. The activation of AMPK has an immense impact on autophagy as it initiates the mammalian Target of Rapamycin (mTOR) pathway, which regulates cellular autophagy in mammalian cells [70]. Furthermore, released calcium can also induce autophagy via the phosphorylation of Protein Kinase CѲ (PKCѲ) [80]. Therefore, ERS is known to be linked to autophagy, and further in-depth studies in elucidating the link are warranted.

## 7. Oxidative Stress

Oxidative stress (OS) is a form of cellular stress and damage due to an imbalance between antioxidant defense mechanisms and Reactive Oxygen Species (ROS) [81]. It commonly arises due to the accumulation of ROS, exerting an oxidative reaction that overpowers the antioxidant activity in the body [81]. A limited number of ROS are commonly found in the body as normal by-products of cellular metabolism [42,82]. However, several factors are known to cause the abnormal production of ROS, including irradiation [83,84]. Upregulation in numerous enzymatic processes, such as mitochondrial respiration, arachidonic acid reaction, Nitric Oxide synthesis, glucose oxidation, and Fenton reactions, also elevates the ROS levels in the body [83,85].

ROS accumulation has detrimental effects on cellular function and homeostasis. Studies have demonstrated that ROS could trigger many cellular signaling pathways and induce damage to DNA, proteins, and cells [81,86]. Persistent exposure to ROS upregulates the production of oxidatively modified nucleic acids, proteins, and lipids, leading to the development of diseases [81,87]. To counteract ROS-induced disturbances, cells develop advanced antioxidant mechanisms to scavenge and reduce ROS production. The antioxidant mechanisms are composed of enzymatic and non-enzymatic reactions and are regulated by superoxide dismutase (SOD), glutathione/glutathione disulfide (GSH/GSSG), and vitamins [18,82,88]. Under normal physiological conditions, the antioxidant mechanisms maintain the redox homeostasis [88]. However, under a disease state, underlying systemic and cellular disorders enhance ROS production and impair the antioxidant mechanisms, causing ROS accumulation and redox imbalance, resulting in OS.

## 8. Vicious Sequence of Events between Endoplasmic Reticulum Stress and Oxidative Stress

The association between ERS and OS is yet to be fully elucidated. Recent studies support the view that ER protein folding pathways are highly correlated with ROS production [4,87]. In ER, redox homeostasis is crucial for the protein folding process and disulfide bond formation [89]. Studies suggest that an alteration in ER-regulated protein folding pathways causes an ROS imbalance and enhances ROS production, indirectly disturbing both ER and redox homeostasis [90]. ROS have a profound effect on the ER protein folding process. It is known that several oxidants, including metal ions, peroxides, and oxidation by-products, can pathologically initiate the UPR [14]. For instance, ketocholesterol, a lipid oxidation by-product, can trigger severe UPR in macrophages and smooth muscle cells; however, this response can be reversed by antioxidants like *N*-acetyl-cysteine [37,91,92].

In the protein folding process, disulfide bond formation is essential for the production of functional, mature, and unique tertiary structured proteins, to stabilize the protein structure. The disulfide bond formation is a reversible process for thiol-disulfide exchange and is catalyzed by numerous ER oxidoreductases, such as ER Protein (ERP) 57 and ERP72 [93]. The protein folding process is highly sensitive to redox homeostasis, hence its redox state is tightly modulated by numerous redox mechanisms such as the GSSG/GSH cycle and Protein Disulfide Isomerase (PDI) reaction (kinetically and thermodynamically) [94].

The GSSG/ GSH cycle has a pivotal role in regulating the protein folding process in the ER. GSH is a non-protein thiol that can undergo oxidation and is converted in GSSG. The balance between GSH and GSSG is crucial in maintaining redox homeostasis. The ratio of GSH to GSSG is approximately 1:1 in the ER lumen; meanwhile, it is approximately 50:1 in the cytoplasm [89]. Hence, it was suggested that a highly oxidized environment optimizes the formation of the disulfide bond [89]. However, this also creates an unfavourable environment for the antioxidant mechanism, as the interaction between GSH and ROS is essential in maintaining ER redox homeostasis [82,89]. Besides, the environment also disrupts the activities of ER-resident proteins, thus elevating the production of misfolded proteins and causing ERS [82,89]. In the protein folding process, disulfide bonding is vital to modify and produce a mature, stable, and functional protein. Issues in disulfide bonding, such as cysteine mispairing and improper disulfide bonding, can produce misfolded proteins [82]. GSH can react and reduce non-native disulfide bonds, allowing misfolded proteins to refold again [95].The proteins may be isomerized for protein refolding, oxidized for disulfide bonding, unfolded by GSH, or undergo ERAD [6,96]. However, the protein refolding process is slow as it requires electron acceptors and is also highly dependent on the redox reaction [6,96]. In the ERS condition, the microenvironment of ER is severely distorted. Large amounts of misfolded proteins are accumulated in the ER. GSH assists in the protein unfolding and refolding process [6,96]. Therefore, when the antioxidant mechanism of GSH is compromised, an increased production of ROS leads to ERS.

**Figure 4:**
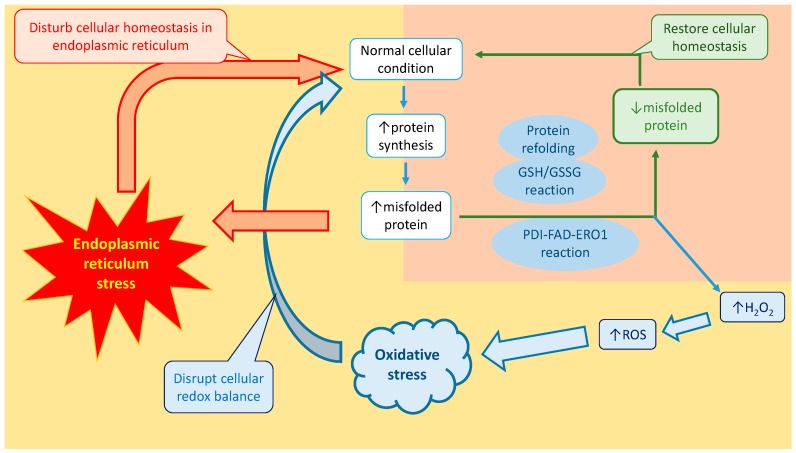
Schematic diagram of the impact of ERS on OS. During ERS, misfolded proteins are accumulated in the ER. GSH helps to reduce the quantities of misfolded proteins by converting non-native disulfide bonds into native disulfide bonds. This event decreases the amount of GSH in the cells. On the other hand, the PDI-FAD-EROI reaction also affects the redox balance in the cells. During the oxidative folding process, PDI receives electrons from the polypeptide substrate chain. FAD, which is recruited by ERO1, conducts a redox reaction on the PDI and produces hydrogen peroxide, which is a type of ROS. As a result, the levels of ROS are increased, while the amount of GSH decreases. Therefore, it disturbs the redox balance and causes OS in the cells. * ERO1, ER Oxidoreductin 1; FAD, Flavin Adenine Dinucleotide; GSSG/GSH, glutathione/glutathione disulfide; PDI, Protein Disulfide Isomerase.

Besides the GSH/GSSG reaction, PDI is also responsible for causing both ERS and OS. Similar to the GSH/GSSG reaction, PDI is a multifunctional oxidoreductase chaperone protein that is involved in the oxidative protein folding process [97]. It is used to catalyze the formation of disulfide bonds [97]. During the oxidative protein folding process, PDI receives two electrons from the polypeptide chain substrate via cysteine residues, and is therefore able to oxidize the polypeptide chain substrate and reduce the PDI protein active sites [97]. However, it has been proposed that other ER resident enzymes also assist PDI in the oxidative folding process. For instance, ER Oxidoreductin 1 (ERO1) was found to play an important role in assisting PDI in the oxidative folding process. After PDI receives the electrons from the polypeptide substrate chain, ERO1 recruits Flavin Adenine Dinucleotide (FAD) to conduct a redox reaction on PDI [97]. The redox reaction transfers the electrons to the oxygen molecules, producing hydrogen peroxide, a type of ROS, and causes OS [97]. In a study by Tu et al., ERO1 null yeast and mice were found to have reduced ROS levels, compared to the control groups [94]. It was reported that about 25% of all the ROS in the in vitro yeast model were produced from an ERO1 redox reaction during the disulfide bond formation [94]. This process enhances the production of ROS and alters the redox balance in the ER. Moreover, the redox imbalance disturbs the protein folding pathway, leads to the upregulation and accumulation of misfolded proteins, and thus induces ERS. Interestingly, several other ER-resident enzymes play a role similar to ERO1, including quiescin sulfhydryl oxidase, peroxiredoxin, and vitamin K epoxide reductase [98]. Figure 4 shows a summary of the connection between ERS and OS.

## 9. Endoplasmic Reticulum Stress and Oxidative Stress in Inflammatory Bowel Disease

Studies on the role of ERS and OS in inflammatory disorders have been extensively conducted. ERS and OS have been implicated by a number of research groups in the pathophysiology of inflammatory conditions like hepatitis, pancreatitis, and inflammatory bowel disease (IBD) [17,99,100,101,102,103].

ERS is known to play a critical role in initiating the inflammatory response. UPR is reported to play a key role in initiating inflammation in the cells. One of the UPR downstream pathways, IRE1 signaling, is known to react with Tumour Necrosis Factor Receptor-associated Factor 2 and initiates c-Jun N-terminal kinase (JNK) and the nuclear factor κ-light-chain-enhancer of activated B cells (NF-κB)-induced inflammation signaling pathways [31,50]. It can also indirectly trigger the release of pro-inflammatory genes by activating their gene promoters [104]. Besides, PERK pathways also accelerate the migration of NF-κB to the nucleus via the phosphorylation of eIF2α and inhibition of the production of the NF-κB inhibitor protein (inhibitor of κ-light-polypeptide-gene-enhancer in B cells, thereby activating the NF-κB-induced inflammatory pathways) [105]. On the other hand, studies have proposed that the ATF6 signaling pathway is also involved in inflammatory diseases. Activated ATF6 is known to enhance the acute phase response in the liver, thus prolonging the inflammation and causing unresolved inflammation [106].

The global burden of IBD, including ulcerative colitis and Crohn’s Disease, is steadily rising [107]. IBD is a chronic relapsing inflammation that causes the formation of ulcers in the gastrointestinal tract, including the large and small intestines. Crohn’s disease can affect the entire gastrointestinal tract, while ulcerative colitis is restricted to the large intestine [107]. Patients suffering from IBD are reported to have ERS in the intestinal epithelial cells such as goblet cells, which produce mucin proteins, such as MUC2 [8,20,108]. Disturbances in the intestinal epithelial cells can damage the intestinal barrier, the mucosal immune system, and homeostasis, thus eliciting an immune response and causing chronic inflammation. Since intestinal epithelial cells consistently produce mucin proteins, they are highly vulnerable to ERS. Elevated levels of ERS markers have been reported in IBD patients, as well as in mice models of colitis compared to the control groups [47,66,68,109,110,111]. Moreover, ENU mutagenesis generated *Winnie* mice with an MUC2 mucin polymorphism leading to misfolded MUC2 in goblet cells, exhibited ERS and IBD symptoms, including elevated misfolded proteins, inflammation in the large intestine, and intestinal epithelial cell disruption [66]. Immunosuppressive agents, such as glucocorticosteroid, has been commonly used in relieving IBD symptoms. Interestingly, dexamethasone (glucocorticoid) treatment in *Winnie* mice reduced the number of misfolded MUC2 proteins and improved protein biosynthesis in the epithelial cells under in vitro and in vivo conditions [112].

Previous studies have proposed that ERS and UPR are involved in the pathophysiology of IBD, causing intestinal inflammation and the disruption of intestinal mucosal homeostasis. Several UPR components have been reported to play a role in the pathophysiology of IBD. For instance, mice with ablated DNA Damage-Inducible Transcript 3, one of the UPR downstream signaling pro-apoptotic proteins, were more protective against dextran sodium sulfate-induced colitis [113]. Besides, IRE1-deficient mice were proven to display a reduction in intestinal epithelial cell apoptosis and mucosal inflammation compared to the control group, after dextran sodium sulfate-induced colitis [69]. IRE1 can cleave and inhibit MUC2 synthesis, and therefore, IRE1 omission is thought to increase MUC2 production [114]. However, the removal of IRE1 could be harmful for IBD patients. IRE1 is crucial when the cells are under an ERS condition, as it decreases the synthesis of MUC2 proteins, resulting in the reduction of the ER workload and thereby relieving the ERS condition. Although UPR is linked to IBD, several UPR components are shown to be vital in maintaining gastrointestinal tract homeostasis. In genetically engineered mouse models, XBP1 knock-out mice displayed disruption in gastrointestinal tract homeostasis, including apoptosis in the paneth cells, a reduction of goblet cells, and ileitis [20,101]. Numerous non-synonymous Single Nucleotide Polymorphisms in XBP1 mRNA coding regions were discovered and reported in IBD patients, and it was proposed that mutations in XBP1 can disrupt gastrointestinal tract homeostasis and cause IBD [68]. Another UPR transducer, Old Astrocytes Specifically Induced Substance (OASIS), is a transcription factor that regulates tissue-specific UPR signalling and is localised in the ER membrane [115,116]. It contains a bZIP domain, a transmembrane domain, and a transcription activation domain [115]. Under an ERS condition, it is cleaved at the transmembrane region, leading to the release of its bZIP domain-containing fragments [115]. These fragment are known to migrate into the nucleus and upregulate the transcription of target genes. Asada and colleagues have discussed the possible role of OASIS in the development of the gastrointestinal tract [117]. It was shown that OASIS-deficient mice exhibit abnormalities in the differentiation of goblet cells in the large intestine [117]. Hence, it was proposed that OASIS might be involved in the terminal differentiation of goblet cells [117]. Moreover, Hino et al. documented that OASIS-deficient mice displayed severe damage in the mucosa of the large intestine and a rapid infiltration of inflammatory cells into the lamina propria [115]. Based on these findings, OASIS is thought to have a function towards the suppression of ERS; however, further studies are warranted to validate these findings.

Interestingly, recent studies have reported the involvement of inflammasomes and ERS in inflammatory pathways. Inflammasome is a signaling protein that consists of NOD-like receptors (NLR), including caspase 1 and NLR Family Pyrin Domain Containing 3 (NLRP3) [118]. Once activated, inflammasome is known to convert pro-caspase 1 into active caspase 1. Active caspase 1 subsequently activates pro-Interleukin (IL) 1β into active IL1β, which is one of the key mediators of the inflammatory response. Inflammasome play a pivotal role in different inflammation-induced autoimmune and metabolic disorders [119,120,121]. It has been hypothesized that ERS is strongly associated with inflammasome during inflammation. For instance, several studies showed that ERS is involved in the activation of pro-IL1β and NLRP3 [122,123,124]. Moreover, ERS was found to activate the NLRP3 inflammasome in the human monocytic cell line (THP-1) [122,125]. Despite the proven association between ERS and inflammasomes, studies are required to understand this correlation in further detail.

Alongside ERS, OS is also known to cause inflammation. ROS can induce inflammation by triggering JNK, Protein Kinase C, Growth factor Tyrosine Kinase Receptor, and Extracellular signal-Regulated Kinase signaling pathways [14]. Several inflammatory transcription factors including NF-κB are redox sensitive and trigger cellular inflammation [126]. IBD patients, as well as mice models of IBD, have elevated levels of ROS in the intestinal mucosal tissue [14]. Mucosal inflammatory pathways are proposed to enhance the production of ROS, such as superoxide and nitric oxide, from intestinal cells, including gut macrophage and epithelial cells [127]. ROS accumulation can damage the intestinal mucosal barrier and induce intestinal inflammation via lipid peroxidation and protein modifications [14]. Therefore, the antioxidant defense mechanisms are necessary for maintaining intestinal homeostasis. For example, genetically modified mouse models with Nuclear Factor (erythroid-derived 2) Like 2 Factor (NRF2)-depletion increased the effect of dextran sodium sulfate to induce colitis [128]. NRF2 is a transcription factor that regulates the antioxidant protein expressions in response to the oxidative damage on the cells. Under the normal condition, NRF2 is retained in the cytoplasm by Kelch-like ECH-Associated Protein 1 (KEAP1) and Cullin3 protein, that degrades NRF2 and prevents the activation of associated downstream signaling pathways. During cellular damage, KEAP1-Cullin3-induced NRF2 degradation is inhibited [129]. Consequently, NRF2 is translocated to the nucleus and binds to the Antioxidant Response Element promoter, upregulating the gene expressions for antioxidant proteins [129]. Overall, OS and ERS seem to play vital roles in IBD pathophysiology.

## 10. Conclusions and Future Directions

Various scientific findings document a functional link between ERS and OS. However, the mechanism behind such a correlation is yet to be fully elucidated. Future studies are warranted to: understand the pathophysiology behind cellular stress-mediated alterations in the protein folding processes, resulting in the production of misfolded proteins; and gain an in-depth understanding of the pathways to determine the precise mechanism(s) of the interactions between ERS and OS signalling. Such findings would make a substantial contribution towards the ERS-OS field of research, leading to the development of new therapeutic interventions for ERS- and OS- associated inflammatory conditions.
